# Action Observation Combined With Virtual Reality Promotes Motor Recovery After Stroke: A Randomized Controlled Trial

**DOI:** 10.1161/STROKEAHA.125.054101

**Published:** 2026-03-18

**Authors:** Antonino Errante, Donatella Saviola, Matteo Cantoni, Katia Iannuzzelli, Chiara Rubertelli, Settimio Ziccarelli, Fabrizio Togni, Marcello Simonini, Carolina Malchiodi, Debora Bertoni, Maria Grazia Inzaghi, Francesca Bozzetti, Annamaria Quarenghi, Paola Quarenghi, Daniele Bosone, Leonardo Fogassi, Gianpietro Salvi, Antonio De Tanti

**Affiliations:** Department of Medicine and Surgery, University of Parma, Italy (A.E., C.R., S.Z., L.F.).; Diagnostic Department, Neuroradiology Unit, University Hospital of Parma, Italy (A.E., F.B.).; Centro Cardinal Ferrari KOS, Parma, Italy (D.S., M.C., K.I., C.M., D. Bertoni, A.D.T.).; Istituto Clinico Quarenghi, San Pellegrino Terme, Italy (F.T., M.S., M.G.I., A.Q., P.Q., D. Bosone, G.S.).

**Keywords:** hemiplegia, mirror neurons, neurological rehabilitation, rehabilitation centers, stroke, upper extremity, virtual reality

## Abstract

**BACKGROUND:**

Action observation treatment and virtual reality are established approaches for upper limb rehabilitation after stroke. This study investigated whether combining action observation with virtual reality improves hand dexterity and upper limb motor recovery compared with virtual reality alone.

**METHODS:**

In this multicenter, assessor-blinded randomized controlled trial conducted in inpatient rehabilitation centers in Italy between January 2022 and September 2024, poststroke adults with hemiplegia (n=48) were assigned to the experimental or to the control group. Both underwent 20 one-hour sessions over 5 weeks. Participants of the experimental group observed goal-directed daily actions before replicating them in virtual reality, whereas participants of the control group viewed nature scenes before performing the same virtual reality tasks without action observation. Motor function was evaluated at baseline, postintervention, and 6-month follow-up using the Box and Block Test, with the primary estimand defined as the between-group difference in change in paretic hand scores. Secondary outcomes included measures of muscle strength, spasticity, global disability, and functional independence.

**RESULTS:**

Both groups showed improvement in Box and Block scores for both hands. However, the experimental group demonstrated a greater gain in paretic hand function, with a between-group difference in change of 7.8 blocks at posttreatment (95% CI, 7.1–7.9) and 10.8 blocks at 6-month follow-up (95% CI, 10.6–10.9). Improvements in the nonparetic hand were comparable between groups. Similar improvements across both groups were observed in secondary outcome measures. A significant treatment×age×time-from-stroke interaction was observed for paretic hand dexterity, indicating that treatment effects varied according to these covariates.

**CONCLUSIONS:**

Action observation combined with virtual reality appears to be more effective than virtual reality alone for promoting upper limb motor recovery after stroke, particularly in enhancing fine motor function of the paretic hand.

**REGISTRATION:**

URL: https://www.clinicaltrials.gov; Unique identifier: NCT05163210.

Stroke is the leading cause of long-term disability in adults,^1^ with >60% of survivors experiencing motor impairments, particularly affecting the upper limb.^2^ It is now widely accepted that poststroke rehabilitation should involve intensive training programs aimed at promoting adaptive plasticity of the sensorimotor system.^3,4^ In line with this principle, various approaches have been developed to stimulate neural reorganization through targeted motor practice. Among these, action observation treatment (AOT) has gained increasing attention over the past 15 years as a promising strategy for enhancing motor recovery.^5^ AOT is based on the systematic observation of goal-directed actions followed by their imitation.^6^ This approach is thought to modulate corticospinal excitability through the activation of the fronto-parietal mirror neuron system.^7–9^ This descending network may play a crucial role in the recovery of fine motor skills after damage to the primary motor cortex or its descending projections.

Building on this neurophysiological basis, several AOT protocols have been developed to harness these mechanisms through the structured observation and imitation of everyday actions.^10–12^ A typical AOT protocol involves practicing everyday actions (eg, grasping a cup to drink) over a period of 2 to 5 weeks, with a frequency of 3 to 5 sessions per week.^6^ During each session, patients are instructed to observe a specific action—presented via short video clips—and then replicate the observed action using the paretic limb.

Nearly 2 decades after the first randomized controlled trial on AOT in patients with stroke by Ertelt et al^12^ numerous studies have confirmed its efficacy, particularly for upper limb rehabilitation.^13,14^ Moreover, AOT has shown benefits in other clinical populations, including patients with Parkinson disease,^15–17^ cerebral palsy,^18–20^ and individuals recovering from orthopedic surgery.^21^

Previous works^6,22^ suggested that AOT is particularly beneficial when intensive motor training is not feasible, for example, due to severe motor impairments, pain, fatigue, or inflammation. However, traditional AOT protocols have some limitations: they are not easily adaptable to the patient’s specific motor abilities, movement patterns, or individual preferences. This lack of personalization can limit the effectiveness and feasibility of AOT in clinical practice.

In recent decades, virtual reality (VR) has emerged as an innovative rehabilitation technique for stroke survivors with hemiplegia.^23–25^ VR-based interventions provide patients with immersive and interactive environments that can be tailored to their motor abilities and therapeutic objectives. Unlike conventional training, VR allows the practice of functional tasks in a safe, motivating, and engaging setting, while offering real-time multisensory feedback that promotes motor learning and neuroplasticity. Moreover, VR systems can be easily adjusted to the individual’s level of impairment and progressively increase task difficulty, thereby enhancing both compliance and rehabilitation outcomes. For these reasons, VR has gained increasing attention as a promising tool to complement or even enhance traditional stroke rehabilitation programs. The largest study to date, involving 376 patients, demonstrated the positive impact of VR-based rehabilitation.^26^ Furthermore, recent systematic reviews have reported significant improvements in motor function and daily living activities after VR-based interventions, compared with conventional therapies.^24^

On these grounds, it has recently been proposed that the combined use of action observation followed by immediate imitation within a VR environment (action observation combined with VR [AO+VR]) may be more effective than conventional VR alone for upper limb rehabilitation in hemiplegic patients.^27,28^ This hypothesis rests on several assumptions. First, the effects of conventional VR are largely attributable to the physical practice of specific exercises, whereas the addition of AOT may prime the corticospinal system before the exercise is actually performed in VR. Second, VR provides highly engaging scenarios that can make AOT exercises more stimulating and less monotonous, thereby reducing the risk of poor adherence to treatment. Third, VR allows exercises to be tailored to the patient’s abilities by lowering task difficulty, thus enabling participation even of individuals with limited residual movement of the paretic limb.

At present, however, high-quality clinical evidence from randomized controlled trials on AO+VR remains scarce—particularly in the context of subacute to chronic stroke rehabilitation. Only a few case reports and pilot studies have investigated similar interventions, yielding mixed results.^28,29^ The aim of the present randomized controlled trial is to evaluate the superiority of the combined AO+VR treatment over standard VR therapy in promoting hand function recovery in subacute to patients with chronic stroke. We hypothesized that adding action observation to VR-based training would enhance sensorimotor facilitation and lead to greater improvements in motor function. Given that action observation elicits bilateral activation of the mirror neuron system, and considering that the VR protocol includes both unimanual and bimanual tasks, we also examined whether the AO+VR intervention might influence both the performance of the paretic and of the nonparetic hand. In addition, a secondary exploratory objective was to investigate whether individual clinical and demographic characteristics—specifically baseline motor function, age, time since stroke onset, and cognitive status—might influence or moderate treatment response.

## Methods

### Research Design and Study Sample

**Figure 1.**
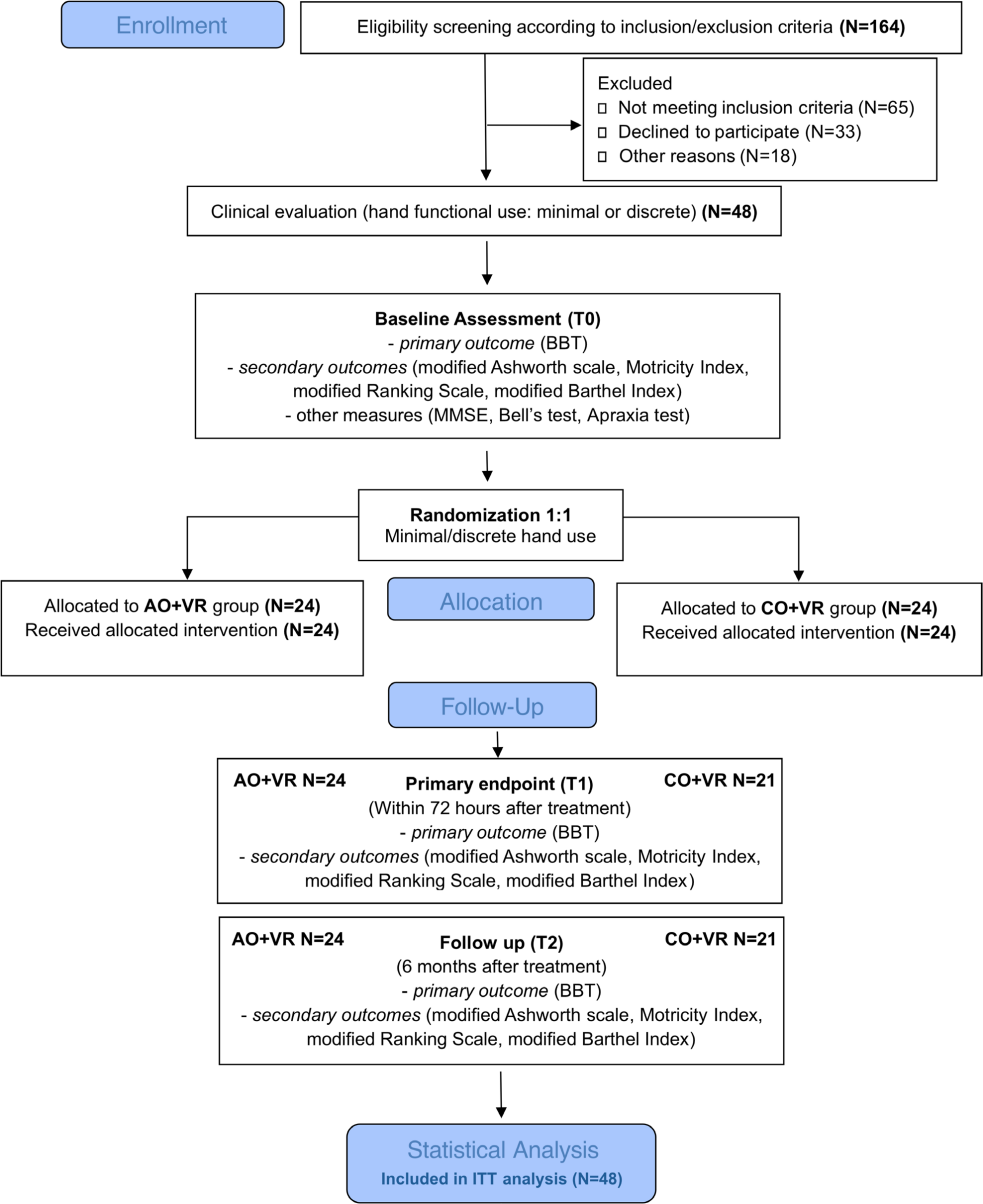
**Flow diagram of the clinical trial according to CONSORT guidelines (Consolidated Standards of Reporting Trials).^31^** All randomized participants were included in the intention-to-treat analysis; missing posttreatment or follow-up data were handled by mean imputation. AO+VR indicates action observation combined with virtual reality; BBT, Box and Block Test; CO+VR, control observation plus virtual reality; ITT, intention-to-treat; and MMSE, Mini-Mental State Examination.

The extended version of the protocol has been fully described elsewhere^30^ and will be briefly summarized here. This study consists in a multicentric randomized allocation concealed (waitlist-controlled) and evaluator-blinded clinical trial with 2 investigative arms, using an intensive rehabilitation program based on action observation followed by imitation in VR (AO+VR; experimental intervention) compared with a control treatment based on observation of control videos followed by action execution in VR (control observation plus VR [CO+VR]; control intervention). This article follows the CONSORT guidelines (Consolidated Standards of Reporting Trials^31^; Figure 1; Table S1). The trial has been approved by the Ethics Committee Area Vasta Emilia Nord (protocol number. 1164/2020/SPER/AOUPR, November 11, 2020) and the ethics committee of Bergamo (Azienda Socio-Sanitaria Territoriale [ASST] Papa Giovanni XXIII, protocol 024/21, May 12, 2021). Before enrollment, written informed consent was obtained from patients or from their caregivers. The data that support the findings of this study are available from the corresponding author on reasonable request.

### Participants

Eligible patients were adults with a clinical diagnosis of stroke (from 3 to 18 months after the acute event, controlled via medical history and patients’ data in their respective rehabilitation centers), resulting in primarily motor symptoms with unilateral upper limb paresis and residual movement ability. Only patients with severe motor symptoms or further severe neuropsychological deficits were not eligible to participate. The multicentric study was performed in 2 clinical centers representing the main Italian venues for stroke rehabilitation based on VR, that is, Centro Cardinal Ferrari (KOS Group, Parma, Italy) and the Clinical Institute Quarenghi (Bergamo, IT). The recruitment and follow-up periods for outcome assessment of benefits and harms ranged from January 2022 to September 2024. The participants were patients admitted to the rehabilitation clinics participating in the study, which also served as the recruitment sites. During the intervention period, all participants were hospitalized and received treatment as inpatients. Follow-up assessments at 6 months were conducted after discharge, when patients were living in the community.

### Inclusion Criteria

1. Primarily motor symptoms with unilateral upper limb paresis (assessed via standard neurological examination).
2. Residual movement ability of the paretic upper limb, assessed by Medical Research Council index=3, active use of the hemiplegic limb, from minimal (mainly for assistance tasks to the preserved limb) to discrete (characterized by coarse manipulation and an inability to perform precision grip).
3. Sufficient cooperation and cognitive understanding to participate in the activities, assessed by the investigator recruiting the patient.

### Exclusion Criteria

1. Presence of severe cognitive impairment (score <20 at Mini-Mental State Examination).
2. Presence of severe forms of unilateral spatial neglect, assessed using the Bells Test (cutoff ≥50%).^32^
3. Presence of severe ideomotor apraxia.^33^
4. Presence of severe anosognosia, assessed by clinical examination.
5. Presence of severe language comprehension deficits, assessed by clinical examination.
6. Presence of severe untreated psychiatric disorders.
7. Sensory impairments hindering participation or not compensated visual deficits of central origin.
8. Drug-resistant epilepsy.

### Setting for VR Rehabilitation

**Figure 2.**
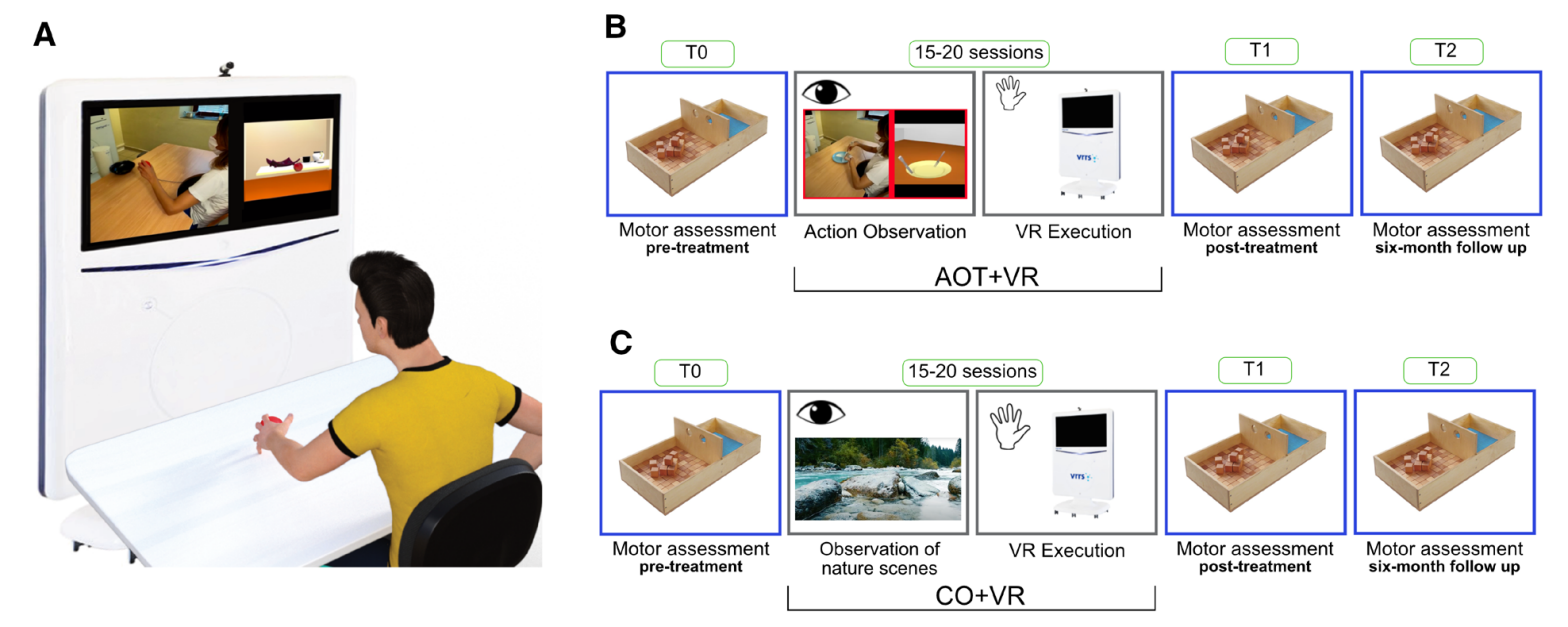
**Experimental setup and study protocol for the action observation combined with virtual reality (AO+VR) and control observation plus VR (CO+VR) interventions. A**, Experimental setting for both AO+VR and CO+VR interventions. **B**, Static frame showing an example of a video clip to be used for the experimental treatment AO+VR based on observation of actions performed by a model, followed by subsequent imitation. **C**, Static frame of a video clip illustrating a natural scene, to be used in the CO+VR control treatment.

Each rehabilitation session was administered in a quiet room, with the patient sitting on a chair and the arms placed on a table. To ensure a suitable range of upper limb movement, the sitting position was adjusted so that the table surface was aligned with the patient’s waist height. For the observation and execution of all exercises in a VR setting, the VR Rehabilitation System (Khymeia Group, Noventa Padovana, Italy) was positioned at 1.5 m distance in front of the patient (Figure 2). For the observation phase, we used the integrated 32-inch high-resolution monitor of the VR Rehabilitation System. This system is used as a clinical routine for the rehabilitation of a wide spectrum of diseases through the numerous rehabilitation modules containing a large number of clinically validated exercises. It also includes a workstation connected to a 3-dimensional motion tracking system (Polhemus Liberty TM, Colchester, VT). The rehabilitation sessions were delivered by 2 physiotherapists and 1 occupational therapist (OT) with >10 years of experience in poststroke upper limb rehabilitation, both with and without technological devices. The centers involved have used VR-based rehabilitation systems, including the Khymeia VR Rehabilitation System, since 2011. All therapists received specific training in the use of the VR platform before the study and participated in the development and refinement of the action observation video materials, ensuring clinical relevance and consistency with the therapeutic goals. During each session, a trained OT or physiotherapist assisted the participants, instructed them to pay close attention to the video clips, and provided encouragement during the imitation tasks.

Concomitant rehabilitation was delivered within the centers’ standardized multidisciplinary poststroke programs and was documented in the patients’ medical records. These programs included physiotherapy, occupational therapy, and task-oriented functional training and were applied equally to participants in both groups according to institutional protocols. Treatment schedules were determined at the ward level and were independent of study allocation. The therapists delivering the VR intervention were not involved in prescribing or modifying conventional rehabilitation.

### Experimental Treatment (AO+VR)

Participants randomized to the experimental group underwent a rehabilitation intervention combining action observation with VR-based motor training (AO+VR). The intervention consisted of 20 sessions (45 minutes each), delivered 4× per week over 5 weeks. A tolerance of up to 5 missed sessions was allowed, resulting in a minimum of 15 and a maximum of 20 completed sessions per participant. Each session began with an action observation phase, during which participants viewed short video clips (≈1.5 minutes in duration) depicting unimanual or bimanual upper limb actions performed by an actor from a lateral perspective. The videos were presented on the VR Rehabilitation System monitor and included both simple and complex motor tasks. The videos consisted exclusively of the visual recording of the action, without any accompanying audio. Immediately after observation, participants were instructed to reproduce the observed actions. During the VR execution phase, participants interacted with real physical objects equipped with inertial sensors for kinematic tracking. By manipulating these objects, patients could simultaneously perceive the movement directly in front of them and observe its corresponding effect within the virtual environment displayed on the VR Rehabilitation System monitor. Each action was imitated at least 3× within a time window of a maximum of 3 minutes. The number of exercises varied depending on the treatment week (see Table S2 for visual description of some exercises). Upper limb movements were tracked using small, lightweight inertial sensors (triaxial accelerometers and gyroscopes) worn by the participants and wirelessly connected to the VR Rehabilitation System Khymeia system via Bluetooth. These sensors allow real-time detection of arm and hand movements and control of virtual objects without restricting joint mobility or hindering functional movement execution. The intervention included 52 distinct exercises, structured in such a way as to increase in complexity across the 5-week period. Exercises were categorized as simple or complex, and as unimanual or bimanual (see Table S3 for the complete list of proposed exercises, organized by week and type). The content was organized weekly as follows:

1. Week 1: No-gravity reaching movements (eg, sliding to reach for targets without arm lifting);
2. Week 2: Simple antigravity actions involving vertical arm movements;
3. Week 3: Complex antigravity actions and bimanual functional tasks (eg, placing toothbrushes in a glass, using a knife and fork);
4. Week 4: Complex bimanual daily life activities (eg, putting on sunglasses, drawing with a ruler); and
5. Week 5: Advanced antigravity tasks, such as obstacle avoidance or path, after using a floating graspable pad.

Each set of exercises for each week was divided into 2 parts: first administered on the first 2 training days of the week and the second on the remaining 2 days. The selection and difficulty of exercises were personalized based on the baseline motor performance of the paretic upper limb. Therapists adjusted the facilitation level of the VRRS system—ranging from 1 to 10—by modifying the sensitivity of the kinematic sensors, enabling participation even for patients with minimal residual hand function. Participants with greater impairment completed no-gravity and simple antigravity tasks, while those with higher motor capacity also performed complex and functional exercises.

### Control Treatment (CO+VR)

Participants randomly assigned to the control group received the same number, frequency, and duration of rehabilitation sessions as those of the experimental group, ensuring an equivalent treatment dose. However, unlike the experimental treatment, participants in the control group were not exposed to action observation before motor training. Instead, each session began with the observation of neutral videos depicting naturalistic scenes without motor content, lasting ≈1.5 minutes. These videos did not contain goal-directed actions/ upper limb movements. After video observation, participants performed motor training in the same VR environment (Khymeia VR Rehabilitation System) as the experimental group. They executed the same set of exercises described for the AO+VR group, including unimanual and bimanual tasks of increasing complexity. They were guided through the exercises via verbal instructions provided by a trained therapist.

The physical setting, equipment, and overall duration of each session were identical to those of the experimental group, including the use of kinematic sensors for 3-dimensional motion tracking and object manipulation in the virtual environment. The key distinction of the control intervention was the absence of the action observation component, making the control condition a pure motor training protocol without an imitation-based component.

### Primary Outcome

The primary outcome was upper limb dexterity, assessed using the Box and Block Test (BBT). The BBT was selected as the primary outcome measure because the study hypothesis was specifically focused on changes in upper limb dexterity induced by the AOT. The BBT directly assesses unilateral manipulation speed and fine gross motor abilities of the hand, and has demonstrated high sensitivity to change in poststroke populations.^34^ The test involves transferring as many as possible of 150 small wooden cubes (side size 25 mm) from one compartment of a wooden box to another one, located on the side, within 60 seconds, using one hand at a time (Figure 2). In addition to evaluating the paretic hand, the BBT performance of the nonparetic hand was also assessed. This exploratory analysis was included because action observation can induce bilateral motor system activation, and the training protocol involved both unimanual and bimanual exercises, potentially affecting dexterity in the nonparetic limb.

In each center, 2 physiotherapists and 1 OT were involved in the study and were assigned either to the role of treating therapist or blinded evaluator. To ensure assessor blinding, the same clinician never performed both roles for the same participant. All evaluators were clinicians with >10 years of experience in poststroke motor assessment, including routine use of the BBT. For the BBT, no minimal clinically important difference has been formally established in stroke populations. Therefore, consistent with previous literature, we adopted the smallest detectable difference (7 blocks^34^) as the benchmark to determine whether observed changes exceeded measurement error and could be considered reliable at the individual level.

Assessments were conducted at 3 time points: T0 (baseline): before the start of the intervention; T1 (posttreatment): within 72 hours after completion of the intervention; and T2 (follow-up): 6 months after the end of the treatment.

### Secondary Outcomes

Secondary outcomes included measures of motor function and functional independence, as well as muscle tone and quality of life. The following standardized clinical scales were used:

modified Ashworth Scale^35^—to assess spasticity;

1. Motricity Index^36^—to quantify motor strength of the upper limb;
2. modified Rankin Scale^37^—to evaluate global disability; and
3. modified Barthel Index^38^—to assess performance in activities of daily living.

These assessments were administered at T0 (baseline) and T1 (posttreatment). In addition, a follow-up assessment (T2) was conducted 6 months after treatment completion to evaluate the long-term retention of the effects.

### Assignment of Intervention

#### Randomization

Participants were randomly assigned to either a control or experimental group with a 1:1 allocation using a computer-generated randomization schedule, stratified by clinical level of hand use (minimal use versus discrete use). In particular, permuted blocks of random sizes were used. The block sizes were not disclosed to ensure concealment. All randomization, sequence generation, and preparation of group allocation materials were performed by an independent researcher, who had no information about the clinical aspects of the trial.

#### Blinding

Participants and their caregivers were informed about the study aims and procedures. Participants were not blinded to group allocation, as the informed consent form described the 2 treatment conditions. In case the patient asked for the presence of the caregiver, the caregiver was seated near the participant but out of her/his view, without interfering with the treatment session. Outcome assessments were administered and scored by a different member of the clinical staff (OT/physiotherapist) who was blinded to group allocation. To ensure assessor blinding, the same clinician never acted as both therapist and evaluator for the same participant. All evaluations were conducted in the same dedicated clinical assessment room within each center, under standardized and controlled conditions. The environment was neutral, quiet, and free from external distractions, ensuring consistency across all assessment time points. Physiotherapists and OTs did not work alongside each other with the same participant during treatment or assessment sessions. For each participant, clinicians delivering the intervention and those performing outcome assessments operated independently and at different times. To further ensure assessor blinding, outcome assessments were conducted in a dedicated clinical room, physically separated from both the conventional rehabilitation areas and the VR rehabilitation rooms. In both centers, these settings were located on different floors of the facilities, preventing incidental contact between assessors and treating therapists and minimizing the risk of unblinding.

### Statistical Analyses

#### Sample Size

At the time of study design, no prior data on the same treatments were available to inform a formal power analysis. Based on prior literature indicating a clinically meaningful 7-point change on the BBT and a common posttest SD of 11.3,^34^ a 2-group comparison with *α*=0.05 and 80% power required 47 participants per group. A 10% attrition rate was included, resulting in a planned total sample of 94 participants. Therefore, a total sample size of 94 participants was initially planned to ensure adequate statistical power to detect clinically meaningful differences between the experimental and control groups.

#### Analyses on Primary and Secondary Outcome Measures

No per-protocol analysis was planned or performed. All analyses followed the intention-to-treat principle. Clinical data were analyzed using IBM SPSS Statistics, version 28.0 (IBM Corp, Armonk, NY). Demographic and clinical data were compared using an independent sample *t* test, Pearson *χ*^2^ test, or Fisher exact test. These comparisons were performed after the normality of variable distributions had been evaluated using the Shapiro-Wilk test. Means and SE of the primary and secondary outcome measures scores at each time point (T0, T1, and T2) were calculated. As first step, differences between groups for all the outcome measures were investigated at baseline (T0) by means of an independent sample *t* test. Then, the primary and secondary outcomes were analyzed using a Generalized Estimating Equations (GEE) model characterized by treatment as a between-subject factor and time (T0, T1, T2) as within-subjects measure. Moreover, time×treatment interaction was also calculated. This methodology considers the autocorrelation intrinsic in repeated measures over time. If the effect identified using GEE analysis was statistically significant (Wald test), a pairwise comparison was performed using the estimated marginal means to identify the 2 measurement points at which a significant difference occurred. The *P* value was adjusted based on Sidak correction for multiple comparisons in post hoc analysis. Missing data for participants who did not complete the T1 or T2 assessments were handled using mean imputation. Specifically, the missing score at a given time point was replaced with the mean score of the participant’s treatment group at that same time point, allowing all randomized participants to be retained in the analysis (intention-to-treat). All statistical analyses were considered significant at *P*<0.05.

To explore the potential influence of individual characteristics on treatment response, additional GEE models were conducted, including baseline motor function, age, time since stroke onset, and cognitive status as covariates. These variables were selected a priori based on their established relevance in poststroke motor recovery. Baseline motor function was entered as a clinical variable reflecting functional hand use level (minimal versus discrete use). This variable was distinct from the primary dexterity outcome. In these models, the same time and treatment factors were retained, and interaction terms between covariates and treatment or time were tested to evaluate whether any of these characteristics acted as potential moderators of the intervention effects. Covariate analyses were exploratory and aimed at identifying potential moderators of treatment response.

## Results

A total of 48 patients were enrolled, out of 164 assessed for eligibility (see CONSORT diagram, Figure 1). The remaining 116 patients were excluded because either they did not meet the inclusion criteria (n=65), declined to participate (n=33), or because of other reasons (n=18). Eligible patients were allocated to the AO+VR group (n=24) and the CO+VR group (n=24). A total of 24 out of 24 participants in the AO+VR group and 21 out of 24 participants in the CO+VR group completed both the T1 and T2 assessments, resulting in 3 drop-outs in the control group. Missing data were due to a worsening of clinical conditions in 3 participants, including accidental trauma and the need for medical treatments unrelated to the study intervention. No drop-outs occurred in the AO+VR group. Demographic information and clinical descriptions about the patients allocated to the experimental and control groups are reported in Table 1. They were matched for age, sex, time from event (days), hand use level, affected side, and treatment duration. All patients completed the assigned intervention program (100% compliance), and no adverse event was detected. Table 2 shows that the experimental and control groups also had similar clinical baseline characteristics. No statistical differences between groups were found in baseline scores for the BBT, Motricity Index, modified Ashworth Scale, modified Rankin Scale, or modified Barthel Index.

**Table 1.**
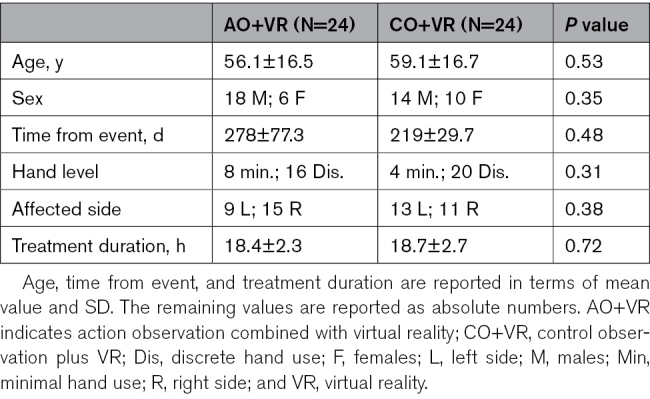
Demographic and Clinical Characteristics of the Participants Allocated to the Experimental and Control Groups

**Table 2.**
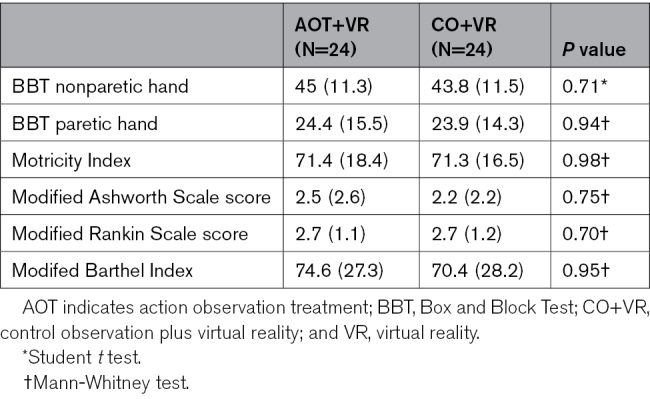
Statistical Comparisons Between Experimental and Control Group Based on Primary and Secondary Outcome Measures at Baseline

**Figure 3.**
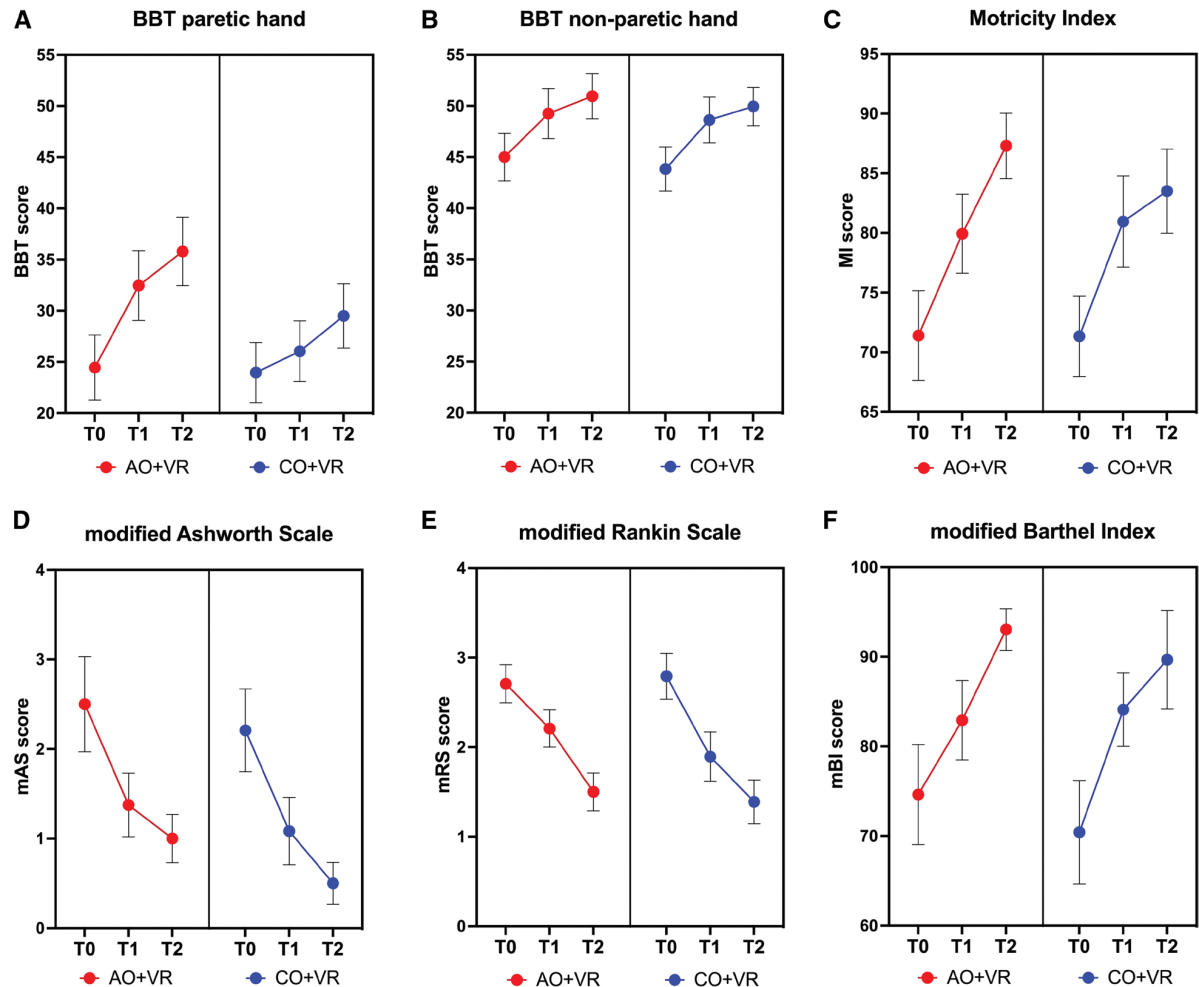
**Mean change over time in the primary and secondary outcome measures.** Score changes in Box and Block Test (BBT) score for the paretic hand (**A**), the non-paretic hand (**B**), Motricity Index (MI; **C**), modified Ashworth Scale (mAS; **D**), modified Rankin Scale (mRS; **E**), and modified Barthel Index (mBI; **F**) are reported separately for action observation treatment (AOT)+virtual reality (VR) group (red dots) and control observation plus virtual reality (CO+VR) group (blue dots), at T0 (before beginning of treatment), T1 (end of treatment, 5 weeks after T0), and T2 (6 months after end of treatment). Error bars represent mean SE (SEM).

Figure 3 shows the within-group change over time and between-group comparison for the primary and secondary outcome measures. These results are presented in the following sections, separately for the 2 types of outcome measures.

### Primary Outcome

The GEE model applied to the primary outcome measure (BBT) revealed a significant time effect for the paretic hand across both groups (GEE, Wald χ^2^=14,688.1; *P*<0.001; Figure 3A). Importantly, a significant time×group interaction was found in this condition (GEE, Wald χ^2^=3263.1; *P*<0.001), indicating a differential treatment effect. Specifically, participants in the experimental group receiving AO+VR showed a significantly greater improvement in motor performance at both T1 and T2 follow-up compared with the control group receiving CO+VR. A significant time effect was also observed for the nonparetic hand. Both the experimental and control groups exhibited comparable improvements over time (GEE, Wald χ^2^=21.1; *P*<0.001; Figure 3B). This finding suggests a generalized, treatment-induced effect extending to the nonparetic upper limb, possibly reflecting mechanisms of secondary hyperspecialization.

Clinically, the improvement in BBT scores for the paretic upper limb from baseline (T0) to posttreatment (T1) revealed a mean increase of 8.0 points (SD=5.1) in the experimental group, compared with a 2.6-point increase (SD=4.0) in the control group. Notably, the improvement observed in the experimental group exceeded the smallest detectable difference established for the BBT (+7 points), indicating a reliable change beyond measurement error, whereas the improvement in the control group remained below this threshold.

### Secondary Outcomes

A significant improvement over time was observed in the Motricity Index (Figure 3C), which assesses muscle strength across 6 major muscle groups—3 in the upper limb (shoulder abduction, elbow flexion, and hand grip) and 3 in the lower limb (hip flexion, knee extension, and ankle dorsiflexion). The GEE model revealed a robust main effect of time (GEE, Wald χ^2^=73.5; *P*<0.001), with no significant time×group interaction and no main effect of group, indicating that both the AO+VR and CO+VR groups improved to a comparable extent. This finding indicates a generalized enhancement in musculoskeletal function after treatment, reflecting improvements not only in distal motor control (as measured by the BBT) but also in proximal muscle strength.

In addition, spasticity—as measured by the modified Ashworth Scale, a widely used clinical tool for quantifying muscle tone in patients with neurological conditions such as stroke—showed a significant reduction over time in both groups (GEE, Wald χ^2^=35.45; *P*<0.001; Figure 3D). No time×group interaction or group effect emerged, indicating that decreases in muscle tone occurred irrespective of treatment allocation. This reduction reflects a beneficial easing of hypertonia, a common impediment to functional recovery in stroke survivors.

To assess the impact of the intervention on activities of daily living, 2 functional outcome measures were considered: the modified Rankin Scale and the modified Barthel Index. The modified Rankin Scale, a widely used clinical scale for quantifying disability and dependence in patients with stroke, showed a significant time effect (GEE, Wald χ^2^=74.5; *P*<0.001; Figure 3E), without interaction or group effects, reflecting reduced disability across both groups. The reduction in modified Rankin Scale scores reflects a shift from moderate to mild disability, indicating improved functional independence. The modified Barthel Index, which evaluates autonomy in performing basic activities of daily living such as feeding, mobility, personal hygiene, and toileting, also showed a significant improvement over time (GEE, Wald χ^2^=23.6; *P*<0.001; Figure 3F), again with no interaction or group differences, indicating comparable gains in activities of daily living independence. On average, patients transitioned from a status of moderate dependence, requiring assistance in several daily tasks, to a state of sufficient functional independence. This suggests a generalization of the intervention’s beneficial effects to real-world functional domains.

### Influence of Age and Time From Event on Treatment Outcome

Exploratory analyses were conducted to examine individual clinical and demographic characteristics that influenced treatment response. Separate GEE models were used to assess the potential moderating role of baseline motor function (minimal versus discrete hand use), age, time-from-stroke-onset, and cognitive status. No significant treatment×covariate interactions were observed for baseline motor function, age, or cognitive status (*P*>0.05, nonsignificant), suggesting that these factors did not moderate the intervention effects.

**Figure 4.**
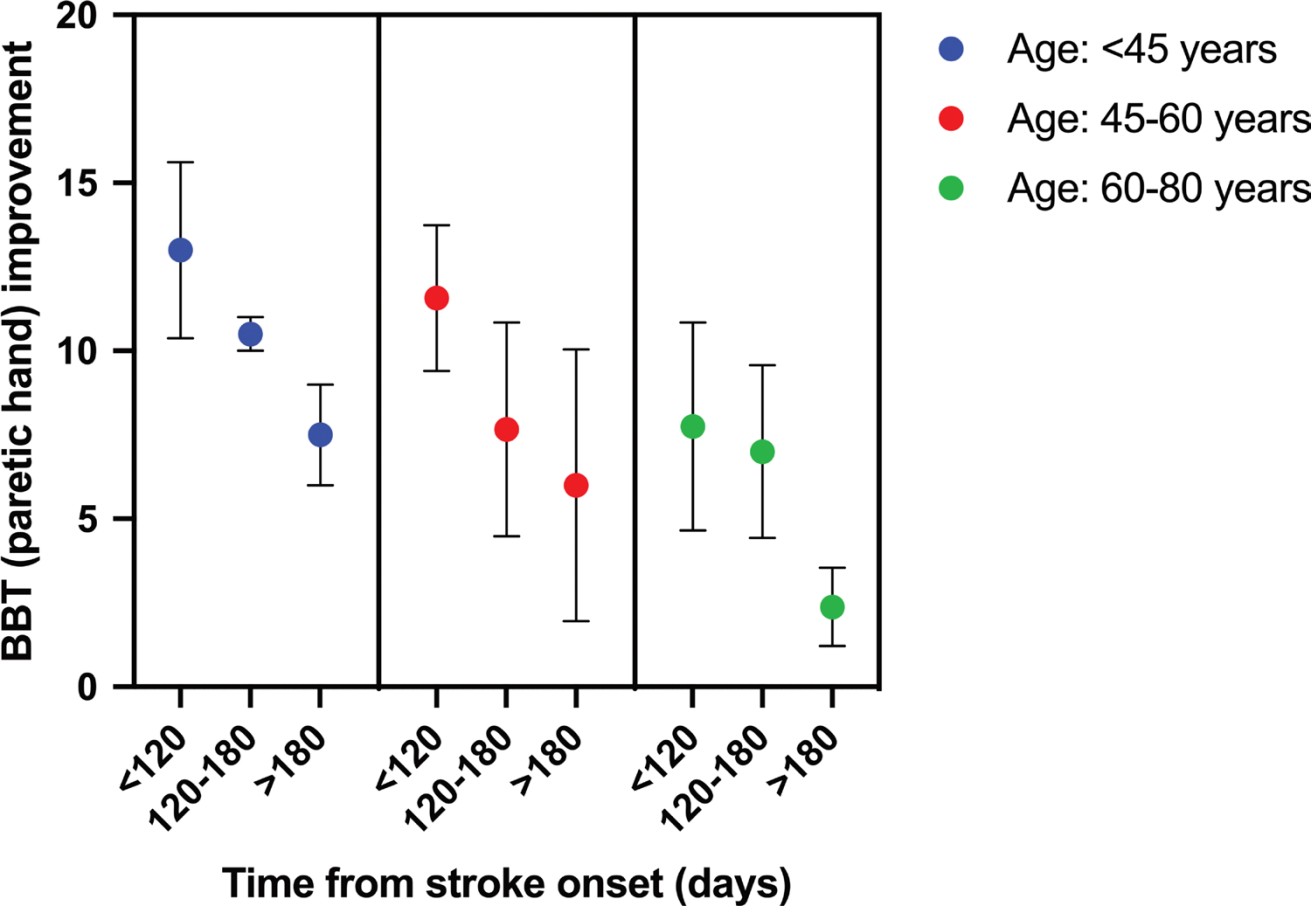
**Interaction effect between age, time-from-stroke-onset, and Box and Block Test (BBT) improvement (paretic hand) emerged from Generalized Estimating Equation analysis.** Error bars represent mean SE (SEM).

A significant age×time-from-stroke interaction was observed for improvement in paretic hand BBT scores across groups (GEE, Wald χ^2^=16.1; *P*<0.001; Figure 4). This pattern indicated greater improvements in participants who were younger and closer to stroke onset. These findings should be interpreted cautiously, given the exploratory nature of the analysis.

## Discussion

The present randomized controlled trial study demonstrates that combining AOT with VR leads to significantly greater improvements in paretic hand function compared with VR alone in hemiplegic patients after stroke. Specifically, the AO+VR group showed a significant improvement in BBT scores for the paretic hand, with changes exceeding the smallest detectable difference. The CO+VR group demonstrated smaller gains that fell below this reliability threshold. Importantly, these benefits persisted at the 6-month follow-up, suggesting that the integration of AOT with VR can promote stable, long-lasting plastic changes in the motor system. Beyond paretic hand recovery, both groups showed improvements in muscle strength, reduced spasticity, and enhanced functional independence. A noteworthy finding was the improvement in BBT scores for the nonparetic hand in both groups, raising new hypotheses about the potential bilateral effects of AOT on motor performance. Finally, interaction analyses revealed that the most pronounced gains were observed in younger participants and in those treated earlier after stroke (<120 days from onset), highlighting the importance of timing and individual patient characteristics in modulating treatment responsiveness.

Several limitations should be acknowledged. First, the study did not include core upper limb measures such as the Fugl-Meyer Assessment or the Action Research Arm Test, which are recommended to enhance standardization and comparability across stroke recovery trials. We selected the BBT as the primary outcome because of its strong validity for assessing manual dexterity and its alignment with the specific goals of the intervention. The sample size, although adequate for detecting differences in the primary outcome, limited more detailed subgroup analyses—particularly those examining the influence of age or time since stroke onset. In addition, the semi-immersive VR environment used in this study may have constrained the magnitude of training effects compared with fully immersive systems, and the absence of direct neurophysiological measures prevents definitive conclusions regarding the mechanisms underlying treatment-induced changes. Future studies should incorporate standardized clinical scales alongside dexterity-focused outcomes, adopt more immersive or adaptive VR-AOT paradigms, and use neuroimaging or neurophysiological tools to clarify the neural substrates of recovery. An additional limitation relates to the clinical setting of the study population. In the Italian rehabilitation system, inpatient stroke rehabilitation may extend beyond the early subacute phase, whereas in other healthcare systems inpatient admission is often limited to the first months after stroke. Therefore, the generalizability of our findings to settings including only early subacute inpatients should be interpreted with caution.

The present findings are consistent with previous literature showing that both AOT^5,10–14,22,39–43^ and VR^24,25^ independently improve upper limb recovery in stroke survivors. AOT, grounded in the activation of the mirror neuron system, facilitates motor learning by enhancing motor resonance through action observation and imitation.^5,44^ VR, in contrast, primarily contributes through high-intensity, repetitive, and task-oriented training in an engaging, interactive environment that can be easily adapted to individual needs. In our study, the combination of AOT and VR resulted in a significant improvement in paretic hand function that surpassed the smallest detectable difference threshold for the BBT, whereas VR alone did not reach this benchmark. This suggests the view that the 2 techniques are not merely additive in their effects, but rather synergistic: AOT may prime the motor system through observational learning, triggering the formation of new motor memories and optimizing the subsequent motor practice in VR.

One of the limitations often reported in relation to AOT application is the low adherence observed in certain categories of patients, particularly those with attentional difficulties or low motivation. In the present study, the integration of VR allowed us to deliver highly customizable exercises, ranging from simple planar sliding movements to antigravity movements, progressively more complex functional actions, and even activities of daily living such as putting on glasses or using a fork and knife during a meal. These exercises, also in the combined AO+VR format, were highly appreciated by patients, not only by younger participants but also by older individuals. Such personalization may enhance adherence to the rehabilitation protocol and, in turn, potentiate the overall effectiveness of AOT, in line with the approach proposed by Franceschini et al,^10^ who emphasized the importance of selecting and adapting exercises that maximally engage the mirror neuron system and are functionally relevant for each patient’s daily life. Another relevant aspect is the imitation of observed actions within a VR setting, which, thanks to the presence of numerous engaging scenarios, makes it possible to offer patients a wide range of rehabilitation tasks while simultaneously adjusting the level of difficulty during the execution phase. Compared with performing tasks in a real-world context, VR also allows to simulate actions that would otherwise be too challenging for the patients, enabling them to experience the possibility of executing these tasks—within the limits of their residual motor repertoire—through feasible, adapted movements.

An unexpected yet clinically intriguing result was the improvement in BBT performance for the nonparetic hand across both groups. Although VR training could plausibly contribute to general motor efficiency through bilateral engagement in the virtual tasks, the involvement of AOT raises interesting questions about bilateral motor facilitation. Previous studies have suggested that action observation activates motor representations bilaterally,^45,46^ possibly through interhemispheric connections and bilateral encoding of observed actions in the premotor and parietal cortex.^47,48^ In line with this view, Lindow et al^49^ demonstrated that callosal tract integrity plays a critical role in BBT performance in stroke survivors, further supporting the notion that dexterity tasks rely on bihemispheric coordination and interhemispheric transfer mechanisms. This raises the possibility that AOT, by enhancing the facilitation of the corticospinal system, may indirectly benefit the contralateral (nonparetic) limb. This phenomenon could reflect secondary hyperspecialization, where improvements in the less-affected limb occur as a product of intensive neural engagement during paretic limb training.^50,51^ Such bilateral facilitation might be particularly relevant in daily life, as improved coordination between limbs is essential for bimanual activities. It would be interesting to further investigate these effects by examining, for example, the improvement of both the paretic and nonparetic limbs after AO+VR rehabilitation in groups of patients with different levels of motor impairment. One possibility is that patients with milder motor deficits may still experience substantial gains in paretic limb function, whereas those with more severe impairment might tend to hyperspecialize the functions of the nonparetic limb. In this scenario, an imaging study using functional connectivity magnetic resonance imaging techniques could appropriately capture and characterize these forms of reorganization within the sensorimotor system.

The interaction between treatment efficacy, age, and time-from-stroke-onset suggests that patient characteristics may influence responsiveness to AO+VR. Younger patients and those treated earlier (<120 days poststroke) tended to show the greatest benefits, consistent with heightened neuroplastic potential in the subacute phase. This finding is consistent with previous literature on the application of AOT in poststroke patients, which has shown greater effectiveness in the subacute period compared with the chronic phase.^22,44,52,53^ However, these analyses were exploratory and should be interpreted cautiously given the sample size. Importantly, meaningful gains were also observed in individuals at later stages poststroke, suggesting that AO+VR retains its value beyond the early recovery window. These findings support the feasibility of integrating AO+VR into clinical practice, building on existing VR-based rehabilitation with high patient adherence.

Overall, this study highlights the clinical potential of combining AOT and VR while acknowledging limitations related to sample size, the semi-immersive VR environment, and the absence of direct neurophysiological measures. Future research should clarify the underlying mechanisms through neuroimaging and neurophysiological approaches—including techniques such as transcranial magnetic stimulation to probe corticospinal excitability and motor system modulation—examine the long-term functional relevance of the bilateral improvements observed here, and explore whether more immersive or adaptive AO+VR systems may further enhance engagement, adherence, and therapeutic efficacy.

## ARTICLE INFORMATION

### Acknowledgments

The authors thank the patients and their caregivers for their participation in the experimental study.

### Author Contributions

Drs Errante, Saviola, Fogassi, and De Tanti designed the research study. Dr Errante, M. Cantoni, K. Iannuzzelli, and Dr Ziccarelli designed the exercises for action observation combined with virtual reality (AO-VR) intervention. Drs Saviola, A. Quarenghi, P. Quarenghi, Bosone, Salvi, and De Tanti recruited the patients. M. Cantoni, K. Iannuzzelli, and F. Togni blindly administered all outcome assessments. D. Bertoni and M.G. Inzaghi were the responsible of neuropsychological assessment. M. Cantoni, K. Iannuzzelli, and F. Togni administered experimental and control interventions. Dr Errante managed the concealed allocation. C. Rubertelli and Dr Bozzetti collaborated in statistical data analysis. Dr Errante took the lead in writing the article, which was drafted by Dr Errante and revised by Dr Saviola, M. Cantoni, Dr Bozzetti, C. Rubertelli, Dr Fogassi, and Dr De Tanti. Consent for publication was obtained by the physiotherapist (K. Iannuzzelli) performing the representative exercises shown in Figure 1 and Table S2. All authors have read and approved the final article.

### Sources of Funding

### Disclosures

None.

### Supplemental Material

CONSORT Checklist

Tables S1–S3

## Supplementary Material


