# Supplementary material for: Action Observation Combined With Virtual Reality Promotes Motor Recovery After Stroke: A Randomized Controlled Trial
Source: Stroke. 2026 Mar 18;57(5):1136–48. doi: 10.1161/STROKEAHA.125.054101 (PMC13117561; doi:10.1161/STROKEAHA.125.054101)
Supplement: Supplementary file 1 [file str-57-1136-s001.pdf]

Action Observation Combined with Virtual Reality Promotes Motor Recovery after stroke: a Randomized Controlled Trial

Supplemental Material

Table S1. CONSORT Checklist. Abbreviations: NA = Not applicable.

Section/topic	No	CONSORT 2025 checklist item description	Reported on page no.
**Title and abstract**			
Title and structured abstract	1a	Identification as a randomised trial	1-2
	1b	Structured summary of the trial design, methods, results, and conclusions	2
**Open science**			
Trial registration	2	Name of trial registry, identifying number (with URL) and date of registration	2
Protocol and statistical analysis plan	3	Where the trial protocol and statistical analysis plan can be accessed	4
Data sharing	4	Where and how the individual de-identified participant data (including data dictionary), statistical code and any other materials can be accessed	17
Funding and conflicts of interest	5a	Sources of funding and other support (eg, supply of drugs), and role of funders in the design, conduct, analysis and reporting of the trial	17
	5b	Financial and other conflicts of interest of the manuscript authors	17
**Introduction**			
Background and rationale	6	Scientific background and rationale	3-4
Objectives	7	Specific objectives related to benefits and harms	4
**Methods**			
Patient and public involvement	8	Details of patient or public involvement in the design, conduct and reporting of the trial	5, 11

Trial design	9	Description of trial design including type of trial (eg, parallel group, crossover), allocation ratio, and framework (eg, superiority, equivalence, non-inferiority, exploratory)	4-10
Changes to trial protocol	10	Important changes to the trial after it commenced including any outcomes or analyses that were not prespecified, with reason	NA
Trial setting	11	Settings (eg, community, hospital) and locations (eg, countries, sites) where the trial was conducted	5
Eligibility criteria	12a	Eligibility criteria for participants	5
	12b	If applicable, eligibility criteria for sites and for individuals delivering the interventions (eg, surgeons, physiotherapists)	NA
Intervention and comparator	13	Intervention and comparator with sufficient details to allow replication. If relevant, where additional materials describing the intervention and comparator (eg, intervention manual) can be accessed	7, 8
Outcomes	14	Prespecified primary and secondary outcomes, including the specific measurement, analysis metric method of aggregation (eg, median, proportion), and time point for each outcome	9, 10
Harms	15	How harms were defined and assessed (eg, systematically, non-systematically)	9
Sample size	16a	How sample size was determined, including all assumptions supporting the sample size calculation	10
	16b	Explanation of any interim analyses and stopping guidelines	NA
Randomisation:			
Sequence generation	17a	Who generated the random allocation sequence and the method used	9
	17b	Type of randomisation and details of any restriction (eg, stratification, blocking and block size)	9
			**Reported on page no.**
Allocation concealment mechanism	18	Mechanism used to implement the random allocation sequence (eg, central computer/telephone; sequentially numbered, opaque, sealed containers), describing any steps to conceal the sequence until interventions were assigned	9
Implementation	19	Whether the personnel who enrolled and those who assigned participants to the interventions had access to the random allocation sequence	9
Blinding	20a	Who was blinded after assignment to interventions (eg, participants, care providers, outcome assessors, data analysts)	9
	20b	If blinded, how blinding was achieved and description of the similarity of interventions	9
Statistical methods	21a	Statistical methods used to compare groups for primary and secondary outcomes, including harms	10
	21b	Definition of who is included in each analysis (eg, all randomised participants), and in which group	10
	21c	How missing data were handled in the analysis	10
	21d	Methods for any additional analyses (eg, subgroup and sensitivity analyses), distinguishing prespecified from post hoc	NA
**Results**			
Participant flow, including flow diagram	22a	For each group, the numbers of participants who were randomly assigned, received intended intervention, and were analysed for the primary outcome	11
	22b	For each group, losses and exclusions after randomisation, together with reasons	11

Recruitment	23a	Dates defining the periods of recruitment and follow-up for outcomes of benefits and harms	5
	23b	If relevant, why the trial ended or was stopped	NA
Intervention and comparator delivery	24a	Intervention and comparator as they were actually administered (eg, where appropriate, who delivered the intervention/comparator, how participants adhered, whether they were delivered as intended (fidelity))	7, 8
	24b	Concomitant care received during the trial for each group	7
Baseline data	25	A table showing baseline demographic and clinical characteristics for each group	
Numbers analysed, outcomes and estimation	26	For each primary and secondary outcome, by group:<br>● the number of participants included in the analysis<br>● the number of participants with available data at the outcome time point<br>● result for each group, and the estimated effect size and its precision (such as 95% confidence interval)<br>● for binary outcomes, presentation of both absolute and relative effect size	11
Harms	27	All harms or unintended events in each group	11-14
Ancillary analyses	28	Any other analyses performed, including subgroup and sensitivity analyses, distinguishing pre-specified from post hoc	11-14
**Discussion**			
Interpretation	29	Interpretation consistent with results, balancing benefits and harms, and considering other relevant evidence	14-16
Limitations	30	Trial limitations, addressing sources of potential bias, imprecision, generalisability, and, if relevant, multiplicity of analyses	14

Table S2. Esempi di esercizi costruiti per il protocollo riabilitativo AO+VR e per il trattamento di controllo CO+VR.

Ex.	**Week 1, No-gravity-simple movements**	
1	Lateral reaching movement	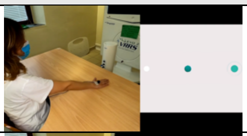
2	Supination/pronation of the hand	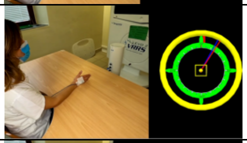
3	Simple trajectory on the horizontal plane	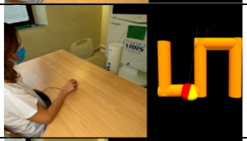
4	Simple trajectory: draw a circle clockwise	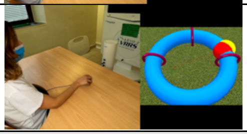
Ex.	**Week 2, simple anti-gravity actions**	
1	Grasping a glass and placing it on a shelf	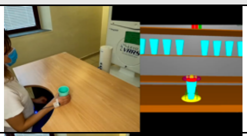
2	Grasping a teapot and move it to the side	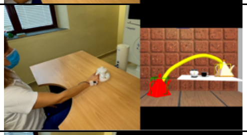
3	Insert the toothbrush in a glass	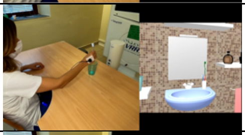
4	Move a book on the chairs with both hands	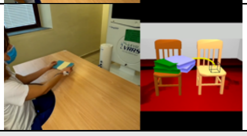
Ex.	**Week 3, complex antigravity actions**	
1	Grasping an object and moving it towards a target (top-down)	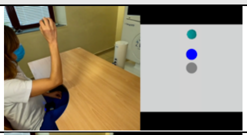
2	Draw a circle with large radius	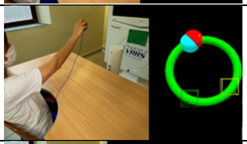
3	Grasp an object and follow a curvilinear path	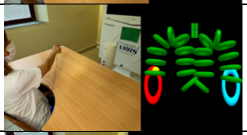
4	Catch water drops with a virtual umbrella	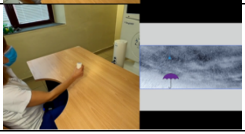

Ex.	**Week 4, complex daily life actions (functional exercises)**	
1	Wearing glasses (bimanual)	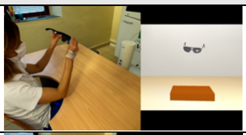
2	Draw a line with the ruler (bimanual)	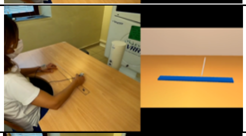
3	Put the toothpaste on the toothbrush (bimanual)	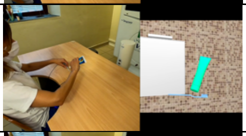
4	Use knife and fork (bimanual)	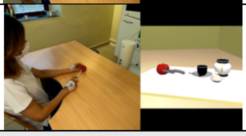
Ex.	**Week 5, advanced anti-gravity tasks**	
1	Copy a drawing using a graspable pad	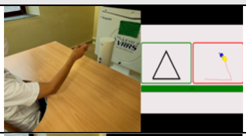
2	Link the points displayed on the screen in the correct order	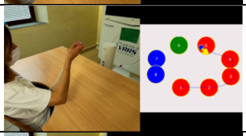
3	Obstacle avoidance: move a ball to the finish without touching the walls	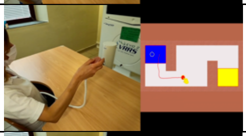
4	Move a ball through the path without leaving it	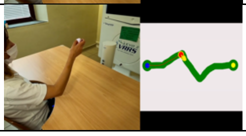

Table S3. Complete list of exercises for the AO + VR and CO + VR treatments.

**Day**	exe. N.	**WEEK 1 "NO GRAVITY" PROTOCOL** The first week was divided into two parts: on days 1–2 (e.g., Monday and Tuesday) patients performed exercises 1 to 7. On days 3–4 (e.g., Wednesday and Thursday) they performed exercises 8 to 14. For each exercise, a difficulty multiplier could be set in order to adapt the task to the patient's manual ability.
**1-2**	1	Backward hand movement
**1-2**	2	Forward hand movement
**1-2**	3	Side-to-side hand movement, right–left
**1-2**	4	Leftward hand movement
**1-2**	5	Rightward hand movement
**1-2**	6	Hand pronation/supination
**1-2**	7	Sliding on a horizontal plane – tracing the number 8
**3-4**	8	Sliding on a horizontal plane – tracing the number 2
**3-4**	9	Sliding on a horizontal plane – path ⎍
**3-4**	10	Sliding on a horizontal plane – path with a ball
**3-4**	11	Forearm supination – compass task
**3-4**	12	Sliding on a horizontal plane – moving a cylinder through a tunnel
**3-4**	13	Sliding on a horizontal plane – drawing a circle (clockwise)

**3-4**	14	Sliding on a horizontal plane – drawing a circle (counterclockwise)

**Day**	exe. N.	**WEEK 2 "SIMPLE ANTIGRAVITY" PROTOCOL** The second week was divided into two parts: on days 1–2 (e.g., Monday and Tuesday) patients performed exercises 1 to 6. On days 3–4 (e.g., Wednesday and Thursday) they performed exercises 7 to 12. For each exercise, a difficulty multiplier could be set in order to adapt the task to the patient's abilities.
**1-2**	15	Short shoulder flexion
**1-2**	16	shoulder abduction and adduction
**1-2**	17	drawing a wavy trajectory
**1-2**	18	wide-range forearm pronation/supination
**1-2**	19	simple reaching (placing a glass on a low shelf)
**1-2**	20	elbow flexion (bringing a glass to the mouth)
**3-4**	21	sliding task – drawing the number 1
**3-4**	22	bimanual task: moving a book across chairs (bimanual)
**3-4**	23	moving a teapot (task with depth)
**3-4**	24	placing a pot on the stove (bimanual)
**3-4**	25	putting a toothbrush in a glass (unimanual)
**3-4**	26	cutting a tomato in half (bimanual)

**Day**	exe. N.	**WEEK 3 "COMPLEX ANTIGRAVITY" PROTOCOL** The third week was divided into two parts: on days 1–2 (e.g., Monday and Tuesday) patients performed exercises 1 to 6. On days 3–4 (e.g., Wednesday and Thursday) they performed exercises 7 to 12. For each exercise, a difficulty multiplier could be set in order to adapt the task to the patient's abilities.
**1-2**	27	forward/backward reaching
**1-2**	28	upward/downward reaching
**1-2**	29	right/left reaching
**1-2**	30	umbrella game - catching falling water drops
**1-2**	31	random reaching (right/left and forward/backward)
**1-2**	32	drawing a large circle in space (shoulder circumduction)
**3-4**	33	follow a serpentine trajectory
**3-4**	34	arm elevation with an object
**3-4**	35	drawing an arc
**3-4**	36	narrow serpentine trajectory
**3-4**	37	medium-range reaching (placing a glass on a mid-height shelf)
**3-4**	38	functional exercise: using knife and fork on a plate (bimanual)

**Day**	exe. N.	**WEEK 4 "COMPLEX FUNCTIONAL AND BIMANUAL ACTIONS" PROTOCOL** During the fourth week, patients performed six unimanual and bimanual exercises on a daily basis. For each exercise, a difficulty multiplier could be set in order to adapt the task to the patient's abilities.
**All**	39	bringing a cellphone to the ear (unimanual)
**All**	40	placing a fork in the sink (unimanual)
**All**	41	placing an apple on a plate (unimanual)
**All**	42	drawing a straight line with a ruler (bimanual)
**All**	43	putting on glasses (bimanual)
**All**	44	applying toothpaste on a toothbrush (bimanual)

**Day**	exe. N.	***WEEK 5 "ADVANCED ANTI-GRAVITY" PROTOCOL*** In the fifth week, the first four exercises (cognitive) could be adapted according to the patient's level of manual ability. For example, in the first exercise, difficulty level 3 was used for more impaired patients, while level 5 was used for more skilled patients. If a patient was unable to perform the cognitive tasks, previous training sessions (e.g., week 3 or week 4) could be repeated. The daily schedule remained identical to that of the corresponding week.

**All**	45-46	Drawing task – reproducing the figure displayed on the screen<br>Levels: 3/5
**All**	47-48	Connecting dots – linking the dots in the correct order<br>Levels: 3/6
**All**	49-50	Obstacle avoidance – guiding the balls to the target without touching the walls<br>Levels: 5/9
**All**	51-52	Path tracking – guiding the ball to the target without leaving the path<br>Levels: 3/9
**All**	38	Functional exercise: using knife and fork on a plate (bimanual)
**All**	44	Functional exercise: applying toothpaste on a toothbrush (bimanual)
